# Pharmacological and Electroceutical Targeting of the Cholinergic Anti-Inflammatory Pathway in Autoimmune Diseases

**DOI:** 10.3390/ph16081089

**Published:** 2023-07-31

**Authors:** Moncef Zouali

**Affiliations:** Graduate Institute of Biomedical Sciences, China Medical University, Taichung 404, Taiwan; moncef.zouali@wanadoo.fr

**Keywords:** electroceutical therapy, inflammation, vagus nerve, neurotransmitters, B lymphocytes, acetylcholine, noradrenaline, lupus, arthritis, diabetes, inflammatory bowel disease, postural orthostatic tachycardia syndrome

## Abstract

Continuous dialogue between the immune system and the brain plays a key homeostatic role in various immune responses to environmental cues. Several functions are under the control of the vagus nerve-based inflammatory reflex, a physiological mechanism through which nerve signals regulate immune functions. In the cholinergic anti-inflammatory pathway, the vagus nerve, its pivotal neurotransmitter acetylcholine, together with the corresponding receptors play a key role in modulating the immune response of mammals. Through communications of peripheral nerves with immune cells, it modulates proliferation and differentiation activities of various immune cell subsets. As a result, this pathway represents a potential target for treating autoimmune diseases characterized by overt inflammation and a decrease in vagal tone. Consistently, converging observations made in both animal models and clinical trials revealed that targeting the cholinergic anti-inflammatory pathway using pharmacologic approaches can provide beneficial effects. In parallel, bioelectronic medicine has recently emerged as an alternative approach to managing systemic inflammation. In several studies, nerve electrostimulation was reported to be clinically relevant in reducing chronic inflammation in autoimmune diseases, including rheumatoid arthritis and diabetes. In the future, these new approaches could represent a major therapeutic strategy for autoimmune and inflammatory diseases.

## 1. Introduction

Homeostatic adaptations and responses to challenging or adverse environmental insults require engagement of both the immune system and the nervous system that interact through several means. Key to these regulations is the ongoing dialogue between the two systems mediated by soluble molecules derived from both neurons and immune cells, and sensory nerves of the autonomic nervous system [1,2]. In the central nervous system (CNS), the interplay between nerve fibers and inflammatory mediators has been well documented. Microglial cells express Toll-like receptors (TLRs), allowing for communication between the immune system and the brain [3]. For example, injection of bacterial lipopolysaccharides (LPS) stimulates nerve signaling in a TLR4-dependent manner [4], and subdiaphragmatic vagotomy mitigates fever triggered by administration of cytokines, namely IL-1β or LPS [5].

In the periphery, cytokines and pathogen-associated molecular patterns stimulate the afferent vagus nerve (VN). The resulting signals navigate through the nucleus tractus solitarius and the dorsal motor nucleus of the VN and are then propagated to the splenic nerve in the celiac plexus. In these vagal interactions with the immune system, the spleen, despite lacking parasympathetic fibers, plays a key role by hosting immune cells that express adrenergic receptors able to interact with norepinephrine (NE) derived from sympathetic nerves [6]. NE conveys multiple cross-talks with sympathetic nerves, namely the alpha- (α-AR) and beta-adrenergic receptor (β-AR) that exert effects in opposite directions. Whereas αARs have stimulatory functions, βARs are inhibitory and exhibit an overall predominant effect. Interaction of NE with β2-adrenergic receptors (β2-ARs) present on lymphocytes causes them to release acetylcholine (ACh), providing a link between NE and immunosuppression (Rosas-Ballina, 2011 #41; Fujii, 2017 #65). ACh binding to the α7 nicotinic ACh receptor (α7nAChR) present on inflammatory cells triggers signal transduction pathways that culminate in the reduction of proinflammatory cytokine production by the spleen, including TNF-α, IL-1β, and IL-6 [7,8,9], but not the anti-inflammatory cytokine IL-10 [10,11]. Overall, the sympathetic nervous system downmodulates immunity, essentially through production of NE, able to inhibit production of proinflammatory cytokines, and to reduce chemotaxis and phagocytosis of neutrophils [12]. Thus, through releasing ACh, the VN exerts anti-inflammatory effects.

**Figure 1:**
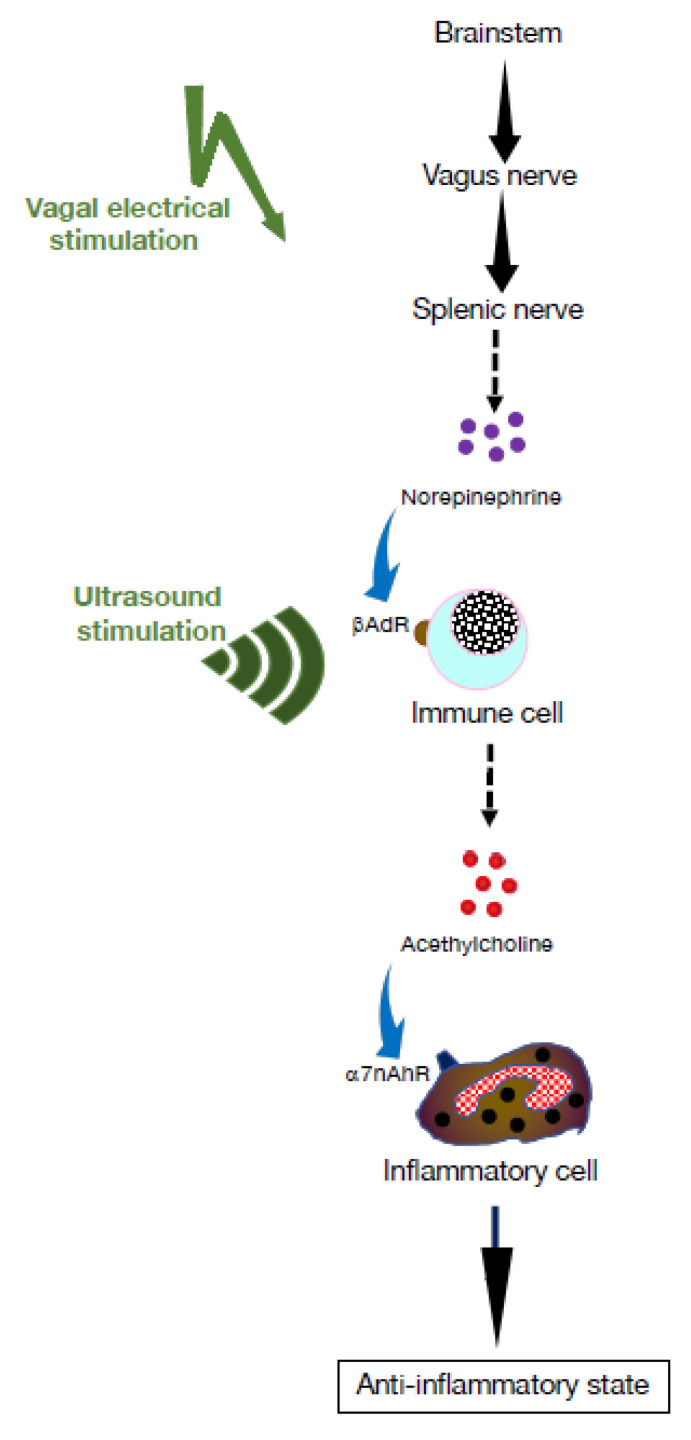
The cholinergic anti-inflammatory pathway. The vagus nerve originates in the brainstem and terminates in the coeliac plexus. From there, the splenic nerve projects to the spleen where its nerve termini locate in proximity of T and B lymphocytes. Following motor signaling through the vagus nerve, norepinephrine is released by nerve termini, which, in turn, triggers release of acetylcholine (ACh) through adrenergic receptors present on lymphocytes. The resulting ACh acts on inflammatory cells expressing α7 nicotinic ACh receptors (α7nAChRs), such as macrophages, and suppresses production of inflammatory cytokines [2,8,15,16].

In pathological conditions, inflammatory signals emanating from peripheral organs reach the CNS via the afferent VN. In turn, the efferent arm of this neuroimmune reflex, termed the cholinergic anti-inflammatory pathway (CAP), is initiated through activation of the splenic nerve, leading to the release of the pivotal neurotransmitter ACh from splenocytes (Figure 1). As a result, the ACh produced activates cholinergic receptors, namely the α7nAChR, which mitigates production of proinflammatory cytokines [13,14]. Converging studies indicate that activation of this pathway mitigates production of proinflammatory cytokines and suppresses systemic inflammation [10,11]. As discussed here, stimulation of this anti-inflammatory pathway can be targeted for therapeutic purposes in several chronic autoimmune diseases (AID), such as rheumatoid arthritis (RA).

## 2. Interfaces between Immune Cells and Nerve Fibers

Communications between the brain and the immune system are mediated by several neuroimmune pathways. Nicotinic ACh receptors are expressed by neurons and neuro-muscular junctions, but also by nonexcitable cells, including skin keratinocytes, respiratory epithelial, vascular endothelial cells, and lymphocytes [17]. Within hours of antigen recognition by immune cells, sympathetic fibers that innervate the parenchyma of lymphoid organs release NE, which, in turn, engages either alpha- or beta-adrenergic receptors (β2AR) present on immune cells, including B cells [18]. Mobilization of lymphocytes into the periphery is regulated by the sympathetic nervous system through catecholamines able to interact with β2AR present on lymphocytes [19], and several studies have revealed that neural reflex mechanisms that stimulate adrenergic receptors of sympathetic nerves control the adaptive arm of immunity [2,20]. For example, NE and β2 agonists can promote antibody production by B cells [6]. Whereas activation of β2-adrenoceptors on monocytes and macrophages exerts immunosuppressive effects, NE plays a β2-mediated anti-inflammatory role by triggering production of ACh by cholinergic B cells in the spleen [6]. Hematopoietic niche cells express β3 adrenergic receptors, which interact with NE released by sympathetic nerve fibers, and this adrenergic signaling impacts antibody production and migration of blood cells from the bone marrow (BM) [21].

**Figure 2:**
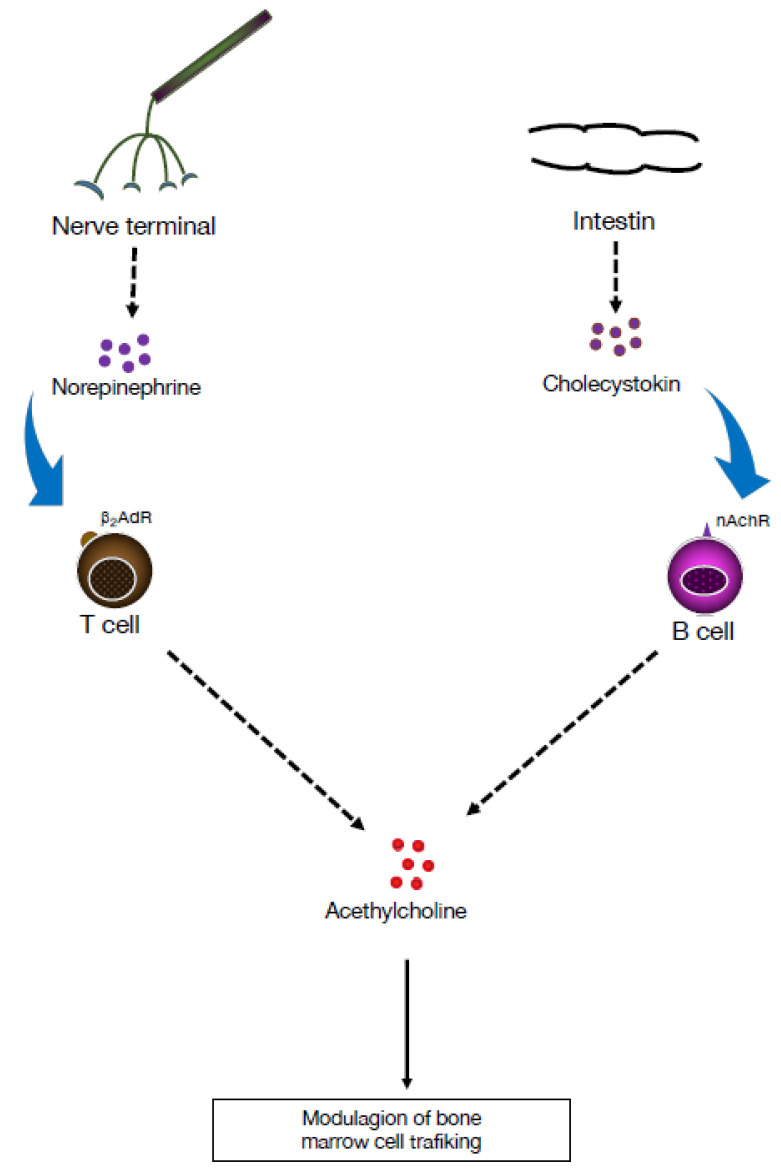
Cell trafficking in the bone marrow is under the influence of neuromediators. Norepinephrine is a catecholamine that functions as a neurotransmitter. Its action on T cells leads to release of acetylcholine (ACh). On the other hand, cholecystokinin is a hormone produced in our small intestine. It stimulates ACh release by B cells through its interaction with β2-adrenernic receptors (β2AdR). The ACh released has an impact on cell trafficking in the bone marrow [24,25].

**Figure 3:**
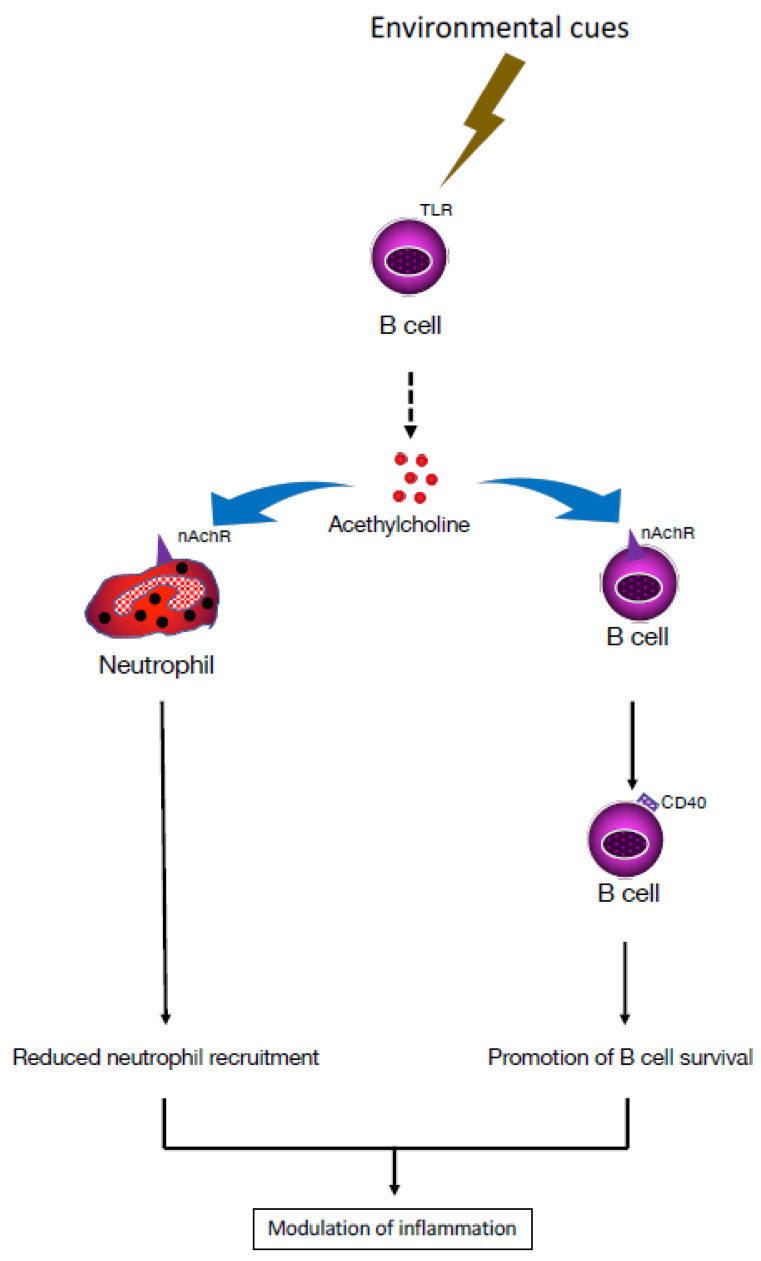
B cell-mediated cholinergic modulation of inflammation. B cells express receptors important for innate immune responses, such as Toll-like receptors (TLR). Likewise, interaction of TLR4 expressed by B cells with pathogen-derived ligands, such as lipopolysaccharides, triggers them to release acetylcholine (ACh), which reduces recruitment of neutrophils and mitigates inflammation. Additionally, through interaction with nicotinic acetylcholine receptors (nAChRs) present on B cells, ACh upregulates CD40 expression on B cells, which promotes their survival in secondary lymphoid organs [3,4,5,22].

Following exposure to a variety of stimuli, including TLRs, B cells express choline acetyltransferase (ChAT), which mediates the synthesis of ACh and/or its receptors [22]. Stimulation of TLRs on B cells upregulates ChAT expression in a transient manner and activates cholinergic activity by enhancing ACh synthesis, resulting in reduction of neutrophil recruitment to the peritoneum during sterile endotoxemia, independently of the VN, indicating that ChAT+ B cells play a role in modulating the local recruitment of neutrophils [23]. In contrast to CD4+ T cells, which produce ACh in response to β2-adrenergic receptor stimulation, B cells are not affected by exposure to NE (Figure 2). However, B cells release ACh in response to cholecystokinin, a neuropeptide that plays a role in the CNS. Together with neurotransmitters emanating from the sympathetic nervous system, i.e., NE and dopamine, ACh regulates trafficking of cells in and out of the BM [24]. Additionally, ACh has been reported to increase the bactericidal activity of neutrophils and macrophages through a muscarinic or nicotinic α4β2 receptor [25]. Therefore, ChAT+ B cells can reduce inflammation in several tissues by reducing endothelial cell adhesion in a muscarinic receptor-dependent manner and by limiting neutrophil recruitment via decreased production of chemokines. These effects indicate that ACh derived from B cells plays a role in regulating innate immunity (Figure 3).

## 3. Cholinergic Functions of B Lymphocytes in Controlling Hematopoiesis 

In early studies, neural signals were demonstrated to control egress of immune cells from the BM [21], and electrical stimulation of the VN was found to regulate the splenic nerve and lead to an arrest of B cell migration and reduced antibody secretion [26]. The fact that increased VN signaling is associated with aggregation of B cells in the marginal zone of the spleen indicates that this anti-inflammatory pathway controls B cell trafficking in the spleen, and that cell migration within secondary lymphoid organs and antibody secretion are under neural control [26]. Thus, neural signaling has the potential to regulate the development of B cell responses by modulating cell trafficking and reorganization of lymphoid architecture.

**Figure 4:**
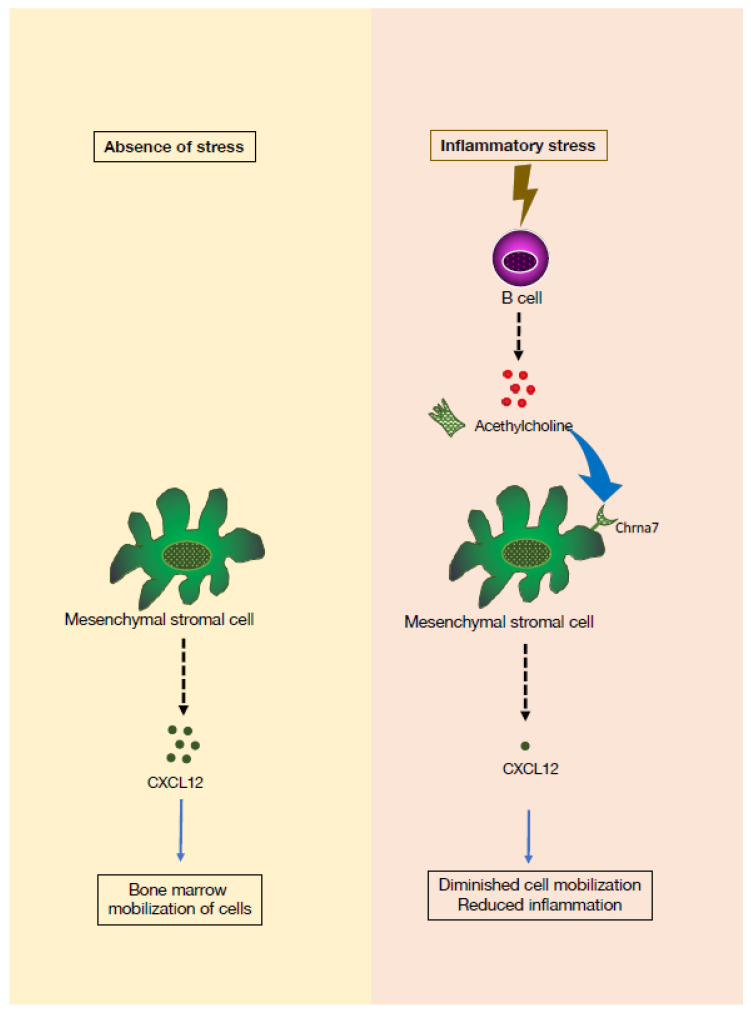
Cholinergic signaling influences lymphocyte mobilization. In normal conditions, B cells release acetylcholine (ACh) in the bone marrow. In response to inflammatory stress, B cell-derived ACh interacts with the cholinergic α7 nicotinic receptor (CHRNA7) present on mesenchymal stromal cells, which triggers expression of mobilization niche factors, such as CXCL12, leading to reduced entry of hematopoietic stem and progenitor cells (HSPC) to cell cycle and differentiation into inflammatory cells of the myeloid lineage [28].

Importantly, B cells express several components of the cholinergic system, including ACh, acetylcholinesterase, ChAT, and muscarinic (mAChRs) and nicotinic (nAChRs) ACh receptors [27]. Recently, studies in both mice and humans demonstrated that augmentation of ACh levels by ACh esterase inhibition reduces hematopoiesis, and that B cells are the primary source of ACh in the BM [28]. In both humans and mice, treatment with an ACh esterase inhibitor (donepezil) that prevents ACh degradation increases the numbers of lymphocytes (Figure 4). Mechanistically, mesenchymal stromal cells sense the presence of ACh in the BM and produce CXCL12, a hematopoietic niche factor that regulates development of hematopoietic stem and progenitor cells (HSPC) in the BM. As a result, proliferation of HSPC is reduced, and hematopoiesis is altered [28]. Identification of BM B lymphocytes as a key cell subset expressing an enzyme required for ACh synthesis, indicates that B cells are an important source of ACh in the BM. The potential of B cells to navigate to various locations of the organism and to interact with a variety of other cell subsets and sense endogenous and environmental cues [29] endows them with important modulatory functions in fine-tuning hematopoiesis. This novel cholinergic function for B lymphocytes in controlling hematopoiesis could have therapeutic implications for the management of inflammatory responses.

## 4. Adrenergic Modulation of the Adaptive Immune Response

**Figure 5:**
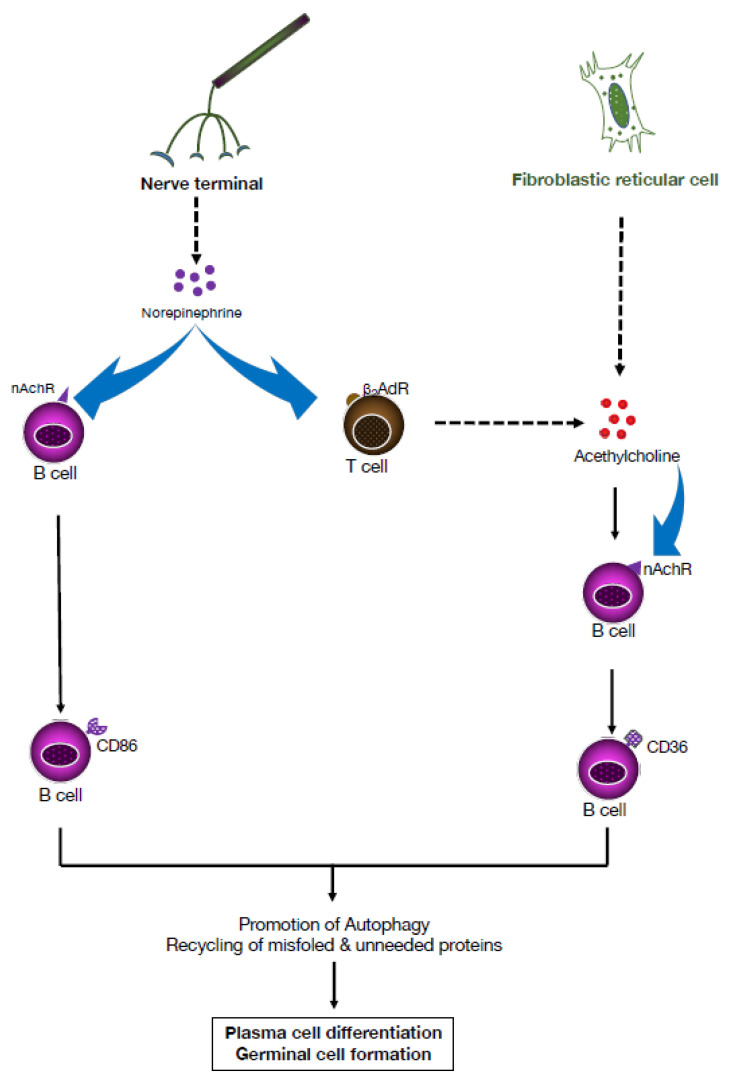
Neuroimmune pathways that promote lymphocyte survival. In secondary lymphoid organs, norepinephrine (NE) released by nerve terminals drives T cells to produce acetylcholine (ACh) via interaction with β-adrenergic receptors (β2AdR) and B cells to upregulate expression of CD86 through nicotinic acetylcholine receptors (nAChRs). CD86 is a costimulatory molecule expressed by B cells and other antigen-presenting cells. It interacts with CD28 for T cell activation and survival. It is usually upregulated following activation of B cells, which are then able to be further activated and, in turn, activate T cells. In the spleen, ACh derived from fibroblastic reticular cells (FRCs) drives upregulated expression of CD36 on B cells, which promotes autophagic functions (recycling misfiled and unneeded proteins) and promotes plasma cell differentiation. These neurotransmitter-mediated events promote lymphocyte survival and germinal center formation. B lymphocyte migration and survival also depend on stromal cells, including fibroblastic reticular cells (FRCs). In the spleen, ACh derived from nerve termini or from FRC upregulates expression of CD36 on B cells, which promotes autophagic functions (recycling misfiled and unneeded proteins) and promotes PC differentiation and GC formation [30,31].

In secondary lymphoid organs, such as the lymph nodes and spleen, lymphocytes interact with components of the local microenvironment, including dendritic cells and fibroblastic reticular cells (FRCs). In addition to direct cell encounters and mechanical cues, soluble factors are present in the microenvironment, namely metabolites, cytokines, chemokines, and neurotransmitters produced by depolarized neurons. In previous B cell studies, ACh binding to nicotinic receptors was reported to promote plasmablast formation [30]. More recently, ACh produced locally by spleen FRCs was demonstrated to trigger enhanced lipid oxidation and an autoimmune response characterized by uncontrolled germinal center formation and expansion of autoantibody-producing plasma cells typical of lupus pathology [31]. Importantly, blockade of either nicotinic or muscarinic ACh receptors mitigated the effect of ACh on B cells, and systemic delivery of ACh worsened lupus-like manifestations in mice. Mechanistically, ACh derived from splenic FRC enhanced lipid oxidation in B cells by upregulating CD36, a scavenger receptor that plays a critical role in B cell responses (Figure 5). In the absence of CD36, mice exhibit reduced plasma cell formation and proliferation, and impaired initiation of autophagy [32].

Further support for the role of adrenergic receptors in modulation of immunity comes from studies of CD86, a transmembrane glycoprotein expressed at low levels primarily on antigen-presenting cells, including B cells. Through binding to CD28 and CTLA-4 expressed on CD4+4 T cells, CD86 acts as a costimulatory molecule that can increase or decrease T cell activation signaling, respectively [33]. CD86 expression on resting B cells can be upregulated in response to engagement with CD40, LPS, IL-4 receptor, or the B cell receptor. Notably, engagement of the β2AR on B cells, by either NE or a selective pharmacologic ligand, regulates lymphocyte activation and CD86 expression (Figure 5).

## 5. Dominance of the Autonomic Sympathetic Nervous System in Autoimmune Diseases

Despite their heterogeneity in terms of inflammatory responses, AID exhibit a common feature, namely the involvement of the autonomic nervous system with its two main components, the sympathetic and parasympathetic branches that jointly regulate immune functions of vertebrate species. A relatively simple means to assess the degree of sympathetic–parasympathetic balance (SPB) in the organism is to determine parameters of heart rate variability (HRV). Remarkably, HRV measures of the SPB revealed an important sympathetic dominance in patients with AID, including thyroiditis, type 1 diabetes (T1D), and RA [34]. Moreover, HRV generally correlates with the severity of the autoimmune response and disease progression in RA, inflammatory bowel disease (IBD), and T1D. This sympathetic dominance in patients with AID could be targeted for therapeutic purposes by pharmacological agents or by bioelectronic medicine. In this latter approach, stimulation can be achieved through the cervical and abdominal vagus nerve, sacral nerve, splenic nerve, and carotid nerve. This therapy has already been tested for treating RA, lupus, Crohn’s disease, and ulcerative colitis.

## 6. Targeting the Cholinergic Anti-Inflammatory Pathway Using Pharmacological Approaches 

**Figure 6:**
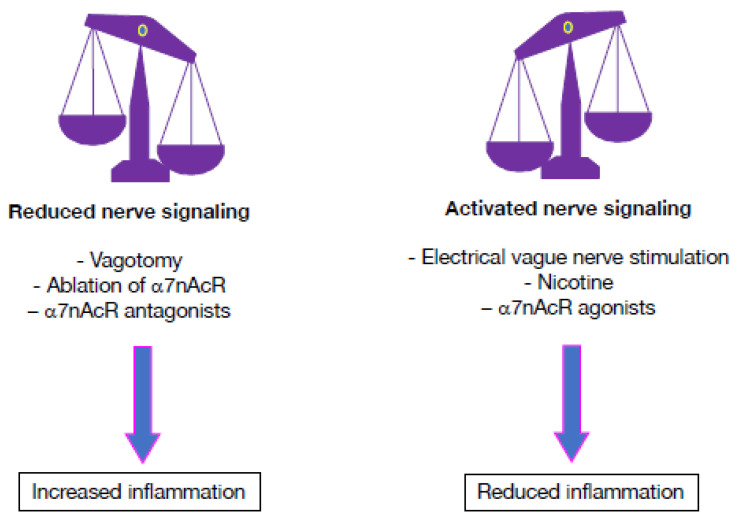
Cholinergic pathways that modulate inflammation. Reduction of vagus nerve signaling by vagotomy, pharmacological agents (antagonists of α7 nicotinic acetylcholine receptors, α7nAChRs) or ablation of α7nAChRs exacerbates inflammatory pathways. In contrast, activation of vagus nerve signaling by electrical stimulation, nicotine, or α7nAChR agonists mitigates inflammation.

Several potential means can be used to activate cholinergic anti-inflammatory processes pharmacologically, including selective agonists or positive allosteric modulators of the α7nAChR to target α7nAChR-positive cells that release proinflammatory cytokines (Figure 6). Studies have demonstrated that nicotinic agonists can reduce the synthesis and production of proinflammatory mediators (IL-1β, TNF-α, IL-6) in a concentration-dependent manner [10,12,13,20]. The direct effect of cholinergic stimulation on isolated immune cell subsets has been addressed in several studies. A cholinergic agonist of nAChRs (nicotine) potently increases IL-12 production by macrophages and dendritic cells in vitro [35,36]. In various models of inflammation, α7nAChR agonists (nicotine, GTS-21, and AR-R17779) were reported to activate α7nAChRs, and the stimulation of the CAP alleviated disease activity [37,38,39]. Reversibly, genetic ablation of the gene encoding α7nAChR triggered production of high plasma levels of TNF-α, and aggravated disease severity of mutant mice [37]. Consistently, specific antagonists of the α7nAChR (α-alpha-bungarotoxin and methyllycaconitine) could deactivate the anti-inflammatory effect of the CAP by blocking α7 receptors in several inflammation models [38].

For therapeutic purposes, it is likely that selective α7 agonists exhibit limited off-target effects. Through their impact on immune cells present in the spleen and other organs, they could activate sympathetic neurons in the CAP and, consequently, promote noradrenergic stimulation of cholinergic cells in the spleen. On the other hand, the efficacy of positive allosteric modulators would depend on the amount of endogenous ACh present or choline provided by reflex activation of cholinergic anti-inflammatory pathways. Activation of the cholinergic pathway by centrally active drugs, including M1 muscarinic receptor agonists (galantamine, or other cholinesterase inhibitors), represents another strategy. These pharmacological agents could promote cholinergic anti-inflammatory responses in the periphery by augmenting cholinergic interactions of immune cells in the spleen and cholinergic nerve–macrophage interactions in the gut and enhancing the stability of ACh in the ganglia.

## 7. Dampening Inflammatory Processes Using Electrical Nervous Stimulation

Studies on the cross-talk between immunology and neurobiology have revealed the importance of reflex neural circuit mechanisms in homeostatic regulation of the innate and adaptive branches of immunity [2]. In the inflammatory reflex model, signals travel through the VN to inhibit production of inflammatory cytokines. The major cholinergic parasympathetic nerve, the VN, innervates several organs, including the gastrointestinal (GI) tract and pancreas. Additionally, nerve termini form synaptic contacts with immune cells in lymphoid tissues, such as the spleen, a key secondary lymphoid organ. However, the VN does not innervate the spleen directly, but terminates in the celiac ganglion. From there, the adrenergic splenic nerve innervates the spleen. Importantly, sympathetic nerves are in close contact with lymphocytes, and this anatomical proximity results in local production of NE, capable of activating β2 receptors on target cells. NE, released within the spleen, acts on lymphocytes, which leads to the release of ACh [8]. In turn, binding of ACh to its receptor (α7 nicotinic acetylcholine receptors (α7nAChRs)) blocks NF-κB nuclear translocation and inhibits inflammasome activation in cells activated by proinflammatory stimulating factors [40,41,42].

Modulation of the activity of peripheral nerves using electric stimulation, termed electroceutical therapy, has been proposed to provide therapeutic benefits in patients with inflammatory diseases who do not respond to pharmaceutical drugs [43,44]. Activation of the CAP can be achieved in situ, pharmacologically through cholinergic receptors, or upstream, by stimulation of the VN, which stimulates both cholinergic receptors of the VN, namely α7nAChR and muscarinic AChR3 (M3AChR), reducing TNF-α, IL-6, and IL-1β release and modulating the inflammatory response [10]. In experimental models of inflammatory syndromes, implantable bioelectronic-based devices capable of targeting the VN were demonstrated to enhance inflammatory reflex signaling, reduce cytokine production, and mitigate disease severity [2,8,10,38,45,46,47]. Remarkably, VNS results in increased plasma concentrations of NE, but does not affect plasma epinephrine.

Optimal preclinical studies on VNS in models of chronic disease require long-term implantation of a VNS device optimized for small animals. Recently, a surgical technique was developed to permanently implant a microcuff electrode onto the mouse cervical VN [48]. The implant successfully inhibits serum TNF-α levels in an acute endotoxemia model. This technique will be useful for further optimal preclinical studies on long-term VN neuromodulation.

For therapeutic purposes, the main disadvantage of stimulating the cervical VN is that this invasive approach can also lead to activation of nerve fibers that cause adverse side effects [49]. Therefore, alternative strategies are being developed. In one approach, stimulation of the auricular branch of the VN could represent a more effective alternative. In a rat LPS acute inflammation model, similar effects were obtained using ultrasound (US) stimulation [50]. US stimulation has the potential to stimulate peripheral nerves and to alleviate inflammation, but the exact underlying mechanisms remain unclear [51,52,53]. It could act through a mechanical or thermal effect that modulates mechanosensitive ion channels of neural tissue membranes, or via a cavitational effect that triggers direct ionic flux, or even voltage-gated ion channels. Additionally, altered expression of genes involved in regulation of the cytoskeleton could affect lymphocyte polarization or migration, and lead to reduced cell infiltration to the inflammatory site and inflammation upon US treatment. In B cells, genes involved in microtubule formation and crosslinking, namely Ssh2, Eml4, and Macf1, were reported to be significantly upregulated, whereas Actb, encoding β-Actin, was significantly downregulated. Since B cells play several roles in inflammatory processes [54], it is tempting to propose US stimulation results in altered expression of genes involved in regulation of the cytoskeleton, which affects cell polarization or migration and leads to a reduced infiltration of B cells and diminished inflammation. It is possible that additional neural signals also regulate ACh release by B cells and shape their response.

## 8. Electroceutical Therapy for Autoimmune Disease

### 8.1. Rheumatoid Arthritis

Standard therapies for RA include pharmacological agents that target inflammatory processes, but a number of patients are not responsive to treatment and suffer from impairments in the quality of life. The inverse relationship between VN activity, as assessed by HRV, and serum levels of inflammatory markers indicates a potential link between insufficient vagal activity and inflammatory processes [55]. There is also evidence that the reduced vagal activity precedes RA development in at-risk patients [38]. Notably, the α7nAChR is detectable in synovial lining cells of RA patients, including macrophages and fibroblast-like synoviocytes (FLS). Therefore, the imbalance in sympathetic/parasympathetic pathways could underly a defective release of ACh capable of binding to the α7nAChR present on inflammatory cells and FLS of the joints. Ex vivo, ACh is capable of significantly reducing the production of cytokines and inhibiting the release of CXCL8, CCL2, CCL3, and CCL5 from IL-1β-stimulated FLS [56]. Consequently, the resulting uncontrolled release of proinflammatory cytokines would promote inflammation in the joint and cartilage erosion [38,57].

Collagen-induced arthritis (CIA) exhibits characteristics reminiscent of the human disease, including inflammation, pannus formation, joint swelling, cartilage destruction, bone erosion, and production of proinflammatory cytokines in the serum. In CIA of the rat, expression of the gene encoding α7nAChR (CHRNA7) is increased, and its inactivation mitigates inflammation [58]. In early studies, treatment with pharmacological agents (nicotine or selective agonists of α7nAChR (AR-R17779, or GTS-21)) reduced clinical signs of arthritis and TNF-α expression in the synovium, improved bone erosion and cartilage loss, and lowered serum levels of proinflammatory cytokines [40]. Reversibly, disease severity was aggravated by vagotomy or genetic deletion of the α7nAChR [37]. 

In experimental models of arthritis, electrical stimulation of the VN reduced disease progression, and vagotomy aggravated disease symptoms by enhancing neutrophil migration (Kanashiro, 2016 #82). In the rat, direct activation of the CAP by VNS reduced inflammation, joint swelling, cytokine production, and synovitis, and mitigated cartilage destruction and periarticular bone resorption [59]. In male Wistar rats, vagal stimulation reduced neutrophil migration and arthritic joint inflammation by activating specific sympathoexcitatory brain nuclei in the locus coeruleus and the paraventricular hypothalamic nucleus [60]. It also led to increased NE levels in the synovial fluid and reduction of synovial inflammatory cytokines. In studies of CIA in female Dark Agouti rats, abdominal VNS reduced disease manifestations, and the treatment diminished systemic levels of RANKL, TNF-α, and histological scores of inflammation and cartilage damage [44]. There was also less infiltration of inflammatory cells. However, this model is not representative of patients suffering from drug-resistant RA. Studies of adjuvant-induced arthritis, which includes more severe bone erosion, would be suitable for validating the utility of abdominal VNS and its potential to reduce off-target effects of cervical stimulation.

Noninvasive US energy delivered to the abdomen of mice during renal ischemic reperfusion injury was reported to reduce inflammation and tissue damage. Remarkably, the anti-inflammatory effects were mediated by T and B lymphocytes [61], and transfer of leukocytes from US-treated spleens to naïve recipient mice could confer protection [62]. In further experiments, daily noninvasive US stimulation that targets the spleen was reported to reduce disease severity in the K/BxN serum-transferred model of inflammatory arthritis [63]. Importantly, both T and B cell populations were involved in the anti-inflammatory pathway, indicating that US stimulation of the spleen has the potential to treat inflammatory diseases. Collectively, these observations suggest that the cholinergic anti-inflammatory pathway can be targeted by direct stimulation of α7 receptors through US and VN stimulations.

In patients suffering from RA, the observation that the α7nAChR plays an important role in the release of inflammatory cytokines and regulation of inflammatory response suggests that vagal electrical stimulation could represent a promising alternative therapy [20]. In a clinical trial, VNS was used to treat RA using implantable vagus nerve electrode cuffs that delivered an electrical current (up to 2.0 mA) to the cervical VN for 60 s one to four times daily [38]. VNS inhibited TNF-α production for up to 84 days and reduced disease severity [38]. Remarkably, suppression of TNF-α release during VNS was observed only when the implantable medical device was functioning. In drug-resistant RA patients, approximately 70% of subjects experienced disease improvement [38].

During cervical VNS, patients may report voice alterations and coughing, and cardiac and respiratory undesirable effects [64]. These side effects are likely due to the fact that the human cervical vagus nerve, which consists of 99% C-fibers at the abdominal level, comprises 80% C-fibers and 20% A- and B-fibers [65]. Stimulation of these latter fibers, characterized by low electrical threshold, can cause activation of the heart, lungs, and larynx [66]. Therefore, more specifically targeting the nerve fiber subset responsible for therapeutic effects would improve efficacy of electroceutical treatment [44]. In a clinical trial, the anti-inflammatory effects of short-term transcutaneous noninvasive VNS (n-VNS) applied to the cervical VN were evaluated in patients with RA [67]. The treatment was well tolerated and provided preliminary support for this therapeutic strategy in patients with RA. However, further investigations using larger placebo-controlled trials are warranted.

An alternative means to stimulating the VN is to apply electrical signals to the cutaneous region supplied by the auricular branch of the VN. In a cohort of RA patients, application of a vibrotactile device to the cymba concha of the external ear reduced peripheral blood production of TNF-α, IL-1β, and IL-6, and alleviated systemic inflammatory responses [68]. The disease attenuation observed persisted for up to seven days in the majority of RA patients. These observations deserve further attention. Prospectively, in parallel to the use of biologics for the treatment of RA, studies on VNS could provide additional benefits to patients affected with this autoimmune disease, such as amelioration of depression and reduction of chronic pain [69,70].

### 8.2. Lupus

Autonomic function tests, including cardiovascular and HRV, have been used to probe autonomic dysfunctions in the autoimmune disease systemic lupus erythematosus (SLE). They revealed differences, when compared to normal subjects [71]. The autonomous nervous system dysfunction, with a prevalence of the sympathetic activity, was associated with a decreased parasympathetic tone and an increase in proinflammatory cytokines [15]. This alteration weakens the VN-mediated anti-inflammatory reflex and, possibly, promotes autoimmunity development. It indicates that the VN is hypoactive. Hence, stimulation of this pathway could alleviate the exacerbated release of inflammatory mediators and reduce inflammation in SLE.

In early studies, nicotine and other cholinergic agonists have been found to significantly reduce the production of proinflammatory mediators through the α7nAChR in models of ischemia perfusion injury [72], but also in experimental models of sepsis and pregnancy-induced hypertension [13,73]. In experimental studies on hypertension-prone lupus mice, administration of nicotine (2 mg/kg/day, subcutaneously) was used to stimulate the CAP at the level of the splenic α7nACh. This treatment reduced hypertension and was associated with lower expression of proinflammatory cytokines in the spleen and the kidney, suggesting that the CAP is impaired in lupus [74]. 

In patients with lupus nephritis, the expression of inflammatory cytokines (TNF-α, IL-1β, and IL-6) is high, and an α7nAChR agonist decreases the levels of these inflammatory mediators. The view that stimulation of the parasympathetic VN using transcutaneous stimulation (tVNS) could be useful in reversing the consequences of autoimmune manifestations has been tested in the human disease. In a randomized, double-blinded, sham-controlled pilot study of 18 patients with lupus, pain, fatigue, and number of swollen joints were significantly reduced following four days of five-minute transcutaneous auricular VNS [75]. However, the effects on other markers of inflammation and disease activity were not documented. Thus, the use of neuromodulation-based bioelectronic medicine for SLE treatment is in its infancy, but further trials could lead to promising options to alleviate lupus symptoms and potentially reverse the disease [16].

### 8.3. Scleroderma

Systemic sclerosis (SSc), also called scleroderma, is a rare systemic autoimmune disorder characterized by typical skin thickening and involvement of several major organs, including the lung, heart, kidney, and GI tract. The autonomic nervous system controls saliva production via the functional M3 muscarinic acetylcholine receptor (M3R) on acinar cells, and deficiency of the M3 mAChR in mice results in hyposalivation. In initial studies, muscarinic agonists (pilocarpine and cevimeline) were reported to stimulate expression of both M1 and M3 receptors in salivary glands and to promote secretory function [76,77]. In addition to inflammatory responses, SSc patients suffer from fatigue, anxiety, and depression, and dysfunction of the autonomous nervous system could account, at least in part, for some clinical manifestations. In a trial of 17 SSc patients with upper GI tract dysfunction, prolonged use of tVNS resulted in normalization of the sympathovagal balance and improvement in GI symptom score [78]. More recently, noninvasive VNS was reported to reduce levels of inflammatory cytokines (IL-6, IL-1β, and TNF-α) in SSc patients [79], suggesting that receptor agonists and VNS could be used to treat this autoimmune disorder.

### 8.4. Diabetes

In type 2 diabetes, stimulation of parasympathetic nerves could modulate β-cells of the pancreas to increase insulin secretion and reduce pathogenesis. In type 1 autoimmune diabetes (T1D), however, β-cells are eventually destroyed by autoreactive lymphocytes. In a diabetes mouse model triggered by streptozotocin, administration of a specific acetylcholinesterase inhibitor prevented hyperglycemia, reduced lymphocyte infiltration into pancreatic islets and preserved the structure and functionality of β-cells, and suppressed production of IL-1β, IL-6, and IL-17 proinflammatory cytokines [80]. The observation that cholinergic stimulation can prevent disease development provides a promising preventive strategy for T1D. Modulation of autoimmune disease severity by administration of AChE inhibitors deserves further attention.

Electrostimulation of autonomic nerves has been demonstrated to block inflammation via the neurotransmitters NE and ACh [20]. However, stimulation of large nerves, such as the VN, can exhibit undesirable side effects on multiple organs. Since autoreactive lymphocytes are activated in pancreatic lymph nodes before migrating to the adjacent pancreas to destroy β-cells in patients with T1D, investigators targeted draining lymph nodes by nerve electrical stimulation. Electrostimulation treatment reduced proliferation of autoreactive lymphocytes and production of proinflammatory cytokines, but also inhibited progression of autoimmune diabetes [81]. All these effects were mediated by activation of β-adrenergic receptors. The fact that pancreatic nerve electrical stimulation mitigates diabetes progression in a mouse model of T1D [81] suggests that electrical stimulation of peripheral nerves for therapeutic purposes, called electroceuticals or bioelectronics, represents a future potential approach for treating AID. Since this electroceutical approach targets the disease triggers rather than the symptoms, it would represent an important shift in T1D therapeutics.

In another hyperglycemic rodent model induced by streptozotocin combined with a high-fat diet, VNS delivered through electrodes implanted at the dorsal subdiaphragmatic vagus resulted in reduction of blood glucose in diabetic rats by enhancing vagal efferent activity and the release of glucagon-like peptide-1 [82]. To gain further insight into the underlying mechanisms, more recent studies applied electrical stimulation to a branch of the VN that only innervates the pancreas [83], thereby abrogating the confounding effects of modulation of liver function, nutrient absorption, and gastric motility on blood glycemia. In a model of streptozotocin-induced diabetes, implantation of a cuff electrode on the pancreatic branch of the VN, followed by electrical stimulation, had protective effects by reducing deficits in Langerhans islet diameter, and ameliorated insulin loss [83]. However, the disease manifestations were monitored only during a snapshot of the disease, which is unlikely to reflect the complex physiopathology of T1D [83], an autoimmune disease characterized by a progressive and continuous attack that ultimately destroys the β-cells. Additionally, there are marked differences in the structure and innervation patterns of the islet between rodents and humans. Therefore, additional studies are required using experimental models that more faithfully mimic the anatomy of human Langerhans islets and disease progression of T1D.

### 8.5. Inflammatory Bowel Diseases

Persistent bowel inflammation in IBD, including ulcerative colitis and Crohn’s disease, is associated with alterations of innate and adaptive immune responses, but also with dysfunction of the enteric nervous system and the gut–brain axis [84]. In Crohn’s disease, drugs that modulate the symptoms by reducing the immune response or inflammation, including corticosteroids and monoclonal antibodies or bioengineered receptors targeting inflammatory cytokines, are not always effective. As a result, a significant proportion of patients are refractory to available conventional treatment options [85].

In addition to acting as an important site for immune surveillance, the GI tract is also an important target for efferent projections from the VN, initially emanating from cholinergic neurons located in the brainstem. Input from the VN to the GI tract can also modulate immune homeostasis in the gut by acting directly through enteric cholinergic neurons, with efferent VN-based mechanisms regulating immune responses through α7nAChR signaling. In experimental colitis, accumulating evidence indicates that nicotinic receptors mediate vagal anti-inflammatory response. In mice suffering from colitis, the acetylcholinesterase inhibitor galantamine and a muscarinic acetylcholine receptor agonist activate the central anti-inflammatory cholinergic pathway, leading to a reduction in mucosal inflammation and proinflammatory cytokine levels [86]. Splenectomy, vagotomy, or splenic neurectomy abrogated this cholinergic anti-inflammatory effect. In another experimental colitis model, treatment with a partial agonist at the α7nAChR (encenicline) reduced cell infiltration into the mucosa and submucosa, including macrophages, neutrophils, and B cells [87]. In the rat, pretreatment with cholinesterase inhibitors (neostigmine or physostigmine) or VNS mitigated colitis severity by acting through the spleen rather than directly in the gut [46,88]. 

In humans, it is intriguing that cigarette smoking has beneficial effects in ulcerative colitis, but aggravating effects in Crohn’s disease [88], probably as a result of the nicotine content of tobacco. Evidence for a beneficial effect of VNS in Crohn’s disease comes from studies of patients with moderate-to-severe disease who manifested clinical, biological, and endoscopic improvement after six months of VNS, and a reduction in inflammation of colonic tissues [47].

### 8.6. Primary Sjögren’s Syndrome

One of the most important symptoms in patients suffering from primary Sjögren’s syndrome (pSS) is chronic fatigue, and autonomic nervous system dysfunction has been reported in these patients [89]. The effects of noninvasive VN activation on immune responses and clinical symptoms of pSS were tested in a cohort of 15 female patients using a handheld, battery powered device that sends electrical signals to activate the VN through the skin and soft tissue of the neck. Patterns of natural killer cells and T cells were altered significantly over the study period [79]. Interestingly, lymphocyte counts at baseline visit correlated with the reduction in fatigue score. In a more recent trial, 40 participants suffering from pSS were randomly assigned to use active or sham noninvasive VNS devices twice daily for 54 days in a double-blind manner [90]. At day 56, significant improvements in three measures of fatigue were observed only in patients using the active device.

### 8.7. Postural Orthostatic Tachycardia Syndrome (POTS)

This condition refers to a heterogeneous autonomic disorder characterized by excessive orthostatic tachycardia in the absence of orthostatic hypotension [91]. Its origin remains under investigation, and a number of underlying physiopathological mechanisms have been proposed, including autoimmune reactions [92]. In support of autoimmune-mediated pathways in POTS is the identification in a subgroup of patients expressing serum antiadrenergic autoantibodies capable of exerting direct agonist and allosteric effects that alter receptor function [93]. Consistently, induction of antibodies with similar functional properties in a rabbit experimental model could trigger a hyperadrenergic POTS phenotype [94]. 

Management of this syndrome remains challenging because pharmacologic agents have limited efficacy or side effects. Recently, a noninvasive approach to stimulating the auricular branch of the VN, termed low-level tragus stimulation (LLTS), was demonstrated to improve cardiovascular autonomic function and suppress inflammation in both animal models and humans [95]. In an established rabbit model of adrenergic autoantibody-induced POTS, transcutaneous LLTS was found to suppress postural tachycardia, improve the sympathovagal balance—including increased acetylcholine secretion—and attenuate the expression of inflammatory cytokines [96]. The observation that LLTS ameliorated autoantibody-induced autonomic dysfunction and inflammation suggests that noninvasive stimulation could offer a novel, effective, safe neuromodulatory therapeutic strategy for patients with POTS, particularly those suffering from the hyperadrenergic subtype.

## 9. Future Prospects

As discussed above, tight interactions between the autonomous nervous system and the immune system can regulate a variety of inflammatory processes, and dysfunctions in this cross-talk could play a role in the onset and/or progression of AID [97,98,99]. In pharmacological approaches, data obtained in preclinical studies open new treatment possibilities for AID using centrally acting cholinergic compounds, namely acetylcholinesterase inhibitors and mAChR ligands [8,100]. Significantly, some AChE inhibitors, including galantamine, are clinically approved drugs for treating chronic neurodegenerative diseases.

Given recent advances in implantable VN stimulators in both humans and experimental models [101,102], electrostimulation would be clinically relevant in reducing chronic inflammation in AID. These electrical stimulation techniques have uncovered powerful nonpharmacologic therapeutic options for chronic inflammatory diseases. Importantly, these devices have been used for decades in patients with refractory epilepsy or depression, without serious immunosuppression or long-term complications [103]. A critical step to move this new field forward experimentally and clinically is to demonstrate that therapy and the underlying mechanism(s) of action are dependent on specific stimulation parameters and targeted activation of specific cell subsets or pathways and are reproducible across preclinical and clinical trials. 

Several approaches for neuromodulation are under investigation to manage neurological conditions, or even in clinical use, including transcranial direct current stimulation, electrical deep brain stimulation, and transcranial magnetic stimulation [104]. In the future, normalizing vagal tone in AID represents a major therapeutic strategy. However, key questions need to be addressed. For example, it will be important to gain further insight into the role of cholinergic B lymphocytes, cells endowed with a potential to navigate throughout the organism and to release copious amounts of ACh.

In electrophysiological stimulations, the specific voltage used and the treatment regimen remain under investigation. Currently, efforts are deployed to design less invasive or noninvasive approaches to VNS, including transvenous vagus stimulation, transcutaneous VNS, and use of a GammaCore device, and clinical efficacy of these approaches remains under investigation. The unique potential of US to provide a nonpharmacological, noninvasive approach to treating inflammatory diseases could open novel opportunities for using US of peripheral nerves and end-organs to manage a variety of AID, such as RA. US stimulation has the potential to stimulate or modulate peripheral nerves [53], but the exact underlying mechanisms remain unclear. It could act through a mechanical or thermal effect that activates or modulates mechanosensitive ion channels of neural tissue membranes, a cavitational effect resulting in direct ionic flux, or even voltage-gated ion channels [52]. For human implementation, the US approach can noninvasively target nerves and cells within the spleen, which could potentially reduce side effects and ameliorate therapy for various AID. Additional experiments are required before large-scale applications in the clinical arena. 

## Data Availability

Data sharing not applicable.

## Abbreviations

α7nAChR: α7 nicotinic acetylcholine receptor; ACh: acetylcholine; AID: autoimmune disease; β2AR: beta-adrenergic receptors; BM: bone marrow; CAP: cholinergic anti-inflammatory pathway; ChAT: acetylcholine transferase; CNS: central nervous system; FRC: fibroblastic reticular cell; IBD: inflammatory bowel disease; GI: gastrointestinal tract; HRV: heart rate variability; LPS: lipopolysaccharide; NE: norepinephrine; RA: rheumatoid arthritis; SSc: systemic sclerosis; T1D: type 1 autoimmune diabetes; TLR: Toll-like receptor; US: ultrasound; VN: vagus nerve; VNS: vagus nerve stimulation.
